# Supplementary material for: Kinetics of Prostate-Specific Antigen after Carbon Ion Radiotherapy for Prostate Cancer
Source: Cancers (Basel). 2020 Mar 4;12(3):589. doi: 10.3390/cancers12030589 (PMC7139713; doi:10.3390/cancers12030589)
Supplement: Supplementary file 1 [file cancers-12-00589-s001.pdf]

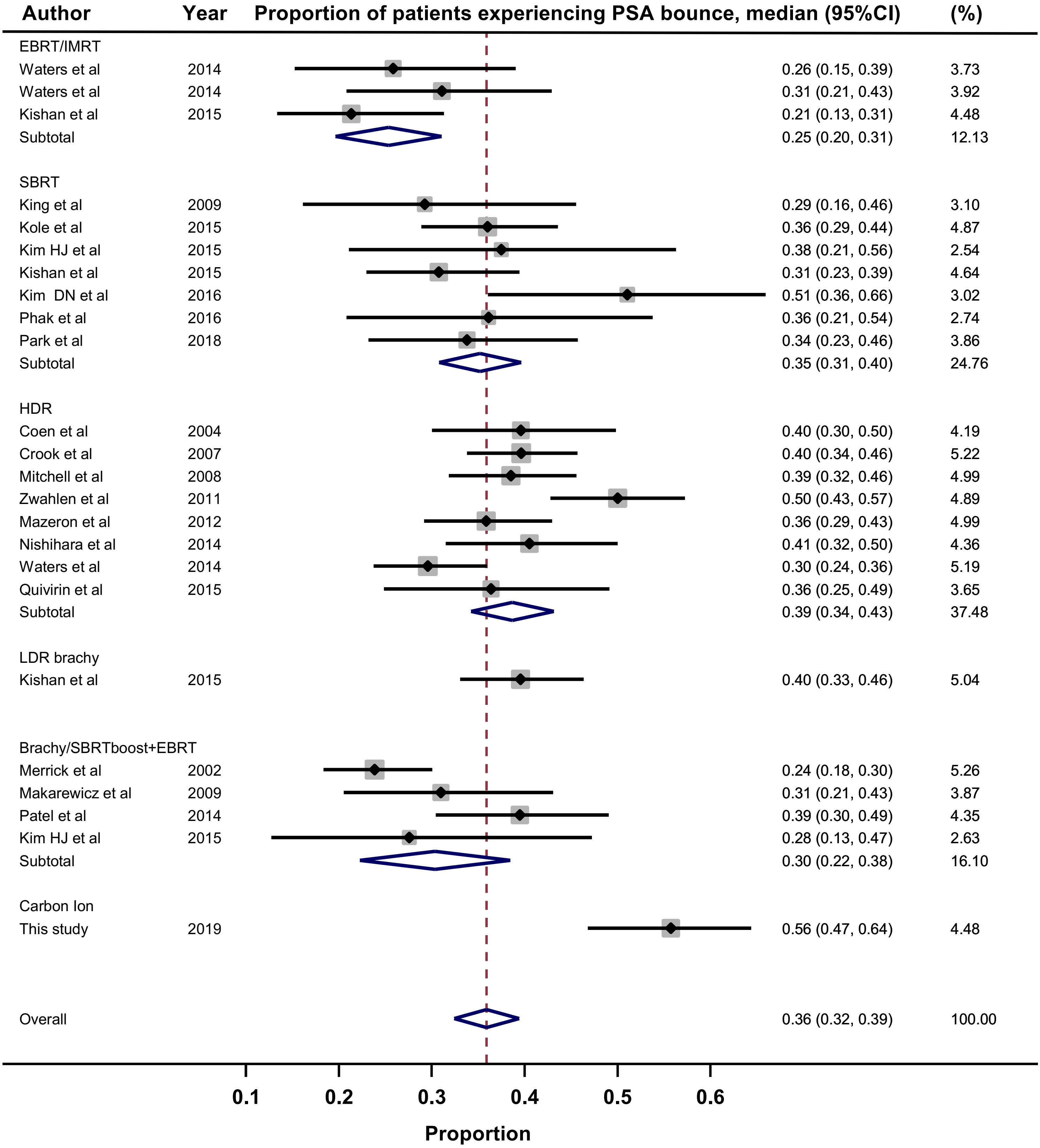

Supplementary Fig. 1. Proportion of patients experiencing PSA bounce after radiotherapy. Previous studies that report PSA bounce after radiotherapy in androgen deprivation therapy-free prostate cancer patients using the cutoff of 0.2 ng/mL were collected by a systematic literature review. PRISMA flow chart was provided as Supplementary Fig. 2.

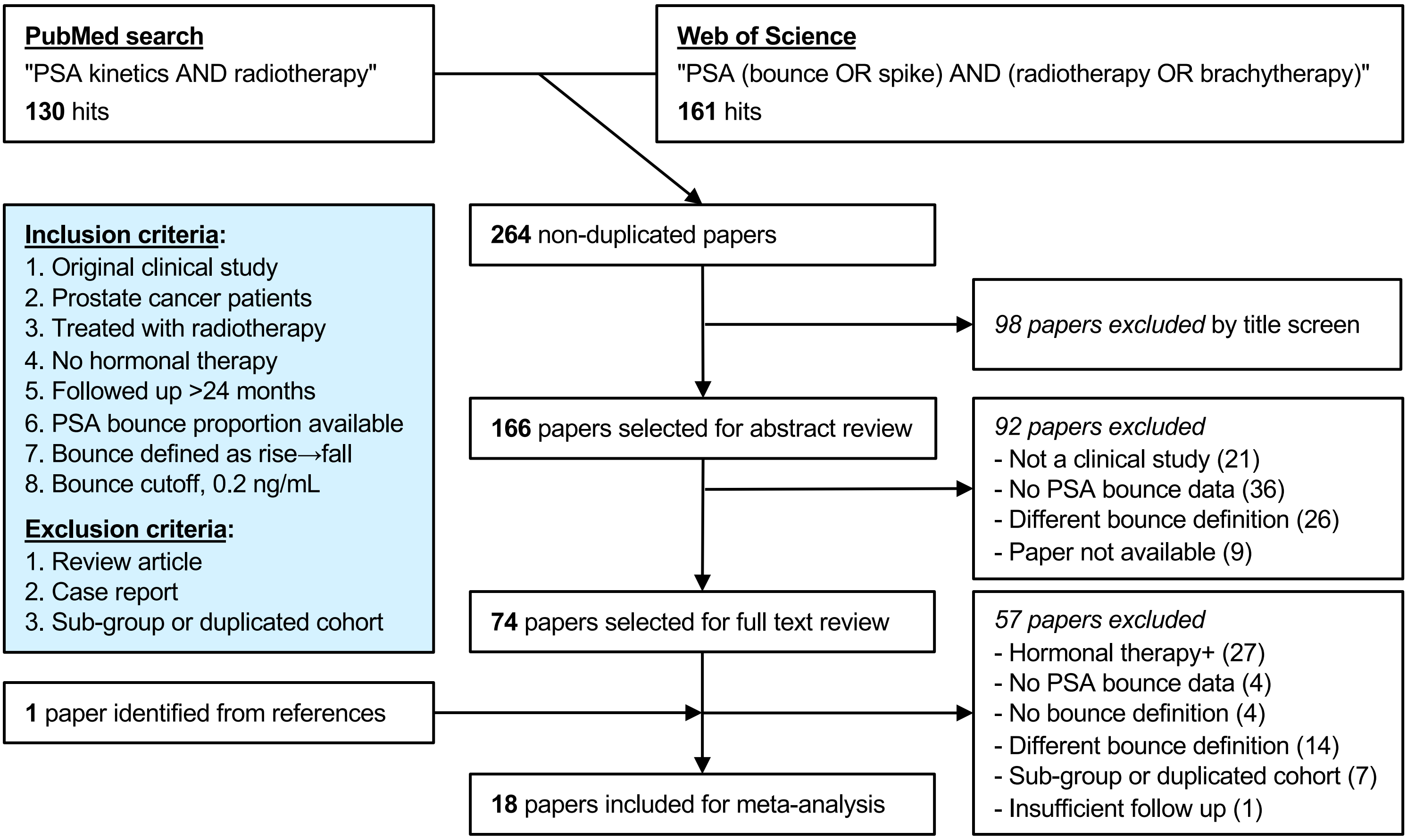

Supplementary Fig. 2. PRISMA flow chart for the systematic literature review on the previous studies that report PSA bounce after radiotherapy in androgen deprivation therapy-free prostate cancer patients using the cutoff of 0.2 ng/mL.
